# Bisphenol A at Environmentally Relevant Doses Inhibits Adiponectin Release from Human Adipose Tissue Explants and Adipocytes

**DOI:** 10.1289/ehp.11537

**Published:** 2008-08-14

**Authors:** Eric R. Hugo, Terry D. Brandebourg, Jessica G. Woo, Jean Loftus, J. Wesley Alexander, Nira Ben-Jonathan

**Affiliations:** 1 Department of Cell and Cancer Biology, University of Cincinnati, Cincinnati, Ohio, USA; 2 Cincinnati Children’s Hospital Medical Center, Cincinnati, Ohio, USA; 3 Christ Hospital, Cincinnati, Ohio, USA; 4 Center for Surgical Weight Loss, University of Cincinnati, Cincinnati, Ohio, USA

**Keywords:** adipocytes, adiponectin, bisphenol A, estradiol, estrogen receptors, estrogen-related receptors, human adipose explants, obesity

## Abstract

**Background:**

The incidence of obesity has risen dramatically over the last few decades. This epidemic may be affected by exposure to xenobiotic chemicals. Bisphenol A (BPA), an endocrine disruptor, is detectable at nanomolar levels in human serum worldwide. Adiponectin is an adipocyte-specific hormone that increases insulin sensitivity and reduces tissue inflammation. Thus, any factor that suppresses adiponectin release could lead to insulin resistance and increased susceptibility to obesity-associated diseases.

**Objectives:**

In this study we aimed to compare *a*) the effects of low doses of BPA and estradiol (E_2_) on adiponectin secretion from human breast, subcutaneous, and visceral adipose explants and mature adipocytes, and *b*) expression of putative estrogen and estrogen-related receptors (ERRs) in these tissues.

**Methods:**

We determined adiponectin levels in conditioned media from adipose explants or adipocytes by enzyme-linked immunosorbant assay. We determined expression of estrogen receptors (ERs) α and β, G-protein–coupled receptor 30 (GPR30), and ERRs α, β, and γ by quantitative real-time polymerase chain reaction.

**Results:**

BPA at 0.1 and 1 nM doses suppressed adiponectin release from all adipose depots examined. Despite substantial variability among patients, BPA was as effective, and often more effective, than equimolar concentrations of E_2_. Adipose tissue expresses similar mRNA levels of *ER*α*, ER*β, and *ERR*γ, and 20- to 30-fold lower levels of *GPR*30, *ERR*α, and *ERR*β.

**Conclusions:**

BPA at environmentally relevant doses inhibits the release of a key adipokine that protects humans from metabolic syndrome. The mechanism by which BPA suppresses adiponectin and the receptors involved remains to be determined.

The incidence of obesity has risen dramatically over the last few decades. Although most attention has focused on high caloric diet and sedentary lifestyle as the root causes, the role of environmental factors is gaining credence. Animal studies suggest that *in utero* or lifetime exposure to xenobiotic chemicals can alter the programming of metabolic homeostasis (Heindel 2003; Newbold et al. 2007). Such chemicals also affect glucose and lipid metabolism as well as adipogenesis in murine adipocytes (Alonso-Magdalena et al. 2006; Masuno et al. 2005). To support the claim that endocrine disruptors may increase the risk of developing obesity-associated disorders, it is critically important to examine their effects on human adipose tissue.

Adiponectin is an adipocyte-specific hormone that protects against metabolic syndrome (Kadowaki et al. 2006). This syndrome is defined by a cluster of conditions that include abdominal obesity, glucose intolerance, hyperinsulinemia, hypertriglyceremia, and hypertension and is associated with increased risk of diabetes and cardiovascular disease (Ritchie and Connell 2007). Serum adiponectin levels are reduced before development of type 2 diabetes, are lower in obese than in lean individuals, and increase after weight loss (Trujillo and Scherer 2005). Because adiponectin is a critical adipokine that increases insulin sensitivity and reduces tissue inflammation (Whitehead et al. 2006), any factor that suppresses its release could lead to insulin resistance and increased susceptibility to development of metabolic syndrome.

Bisphenol A (BPA), a monomer of poly-carbonate plastics, is one of the highest-volume chemicals in commerce. Polycarbonates are used in numerous consumer products, including food and water containers, baby bottles, linings of metal food and beverage cans, medical tubing, epoxy resins, and dental fillings. Small amounts of BPA can migrate from polymers to food or water, especially when heated (Le et al. 2008). Dozen of studies have documented widespread human exposure to BPA. Levels of BPA ranging from 0.3 to 5 ng/mL (~ 1–20 nM) are present in adult and fetal human plasma, urine, and breast milk (reviewed by Welshons et al. 2006). BPA, a lipophilic compound, can accumulate in fat, with detectable levels found in 50% of breast adipose tissue samples from women (Fernandez et al. 2007).

BPA has been reported to alter several metabolic functions (Alonso-Magdalena et al. 2005, 2006; Masuno et al. 2005; Sakurai et al. 2004). However, a major issue relates to the micromolar doses of BPA used in some of these studies. Until BPA is proven active at environmentally relevant concentrations (the low nanomolar range), it is not certain that it poses risks to human health. Moreover, BPA often exhibits a lack of linear dose-dependent relationship, showing instead U-shaped or inverted U-shaped curves. Consequently, extrapolation from an action, or lack of action, of BPA at high doses to its presumed bioactivity at low doses is unwarranted.

The mechanism by which BPA exerts its biological actions is enigmatic. Although BPA binds both estrogen receptors (ERs) α and β (Kuiper et al. 1998), its binding affinity is several orders of magnitude lower than that of estradiol (E_2_), suggesting that it should mimic or compete with endogenous estrogens only at the micromolar range. Yet, BPA at nanomolar doses often displays stronger estrogen-like activities than E_2_ itself. Several speculations have been proposed to reconcile this discrepancy: *a*) BPA binds differently within the ligand-binding domain of ERα or ERβ and recruits dissimilar coregulators (Safe et al. 2002); *b*) BPA elicits rapid responses by binding to membrane-anchored ERs (Watson et al. 2005), an as-yet-unidentified non-classical membrane ER (ncmER; Alonso-Magdalena et al. 2005), or G-protein–coupled receptor 30 (GPR30; Thomas and Dong 2006); and *c*) BPA binds to estrogen-related receptor γ (ERRγ), an orphan nuclear receptor belonging to the ERR family of receptors that do not directly bind E_2_ (Ariazi and Jordan 2006). BPA was recently reported to bind at high affinity to ERRγ (Okada et al. 2008).

The objectives of the present study were to *a*) compare the effects of low doses of BPA and E_2_ on adiponectin secretion from human breast, subcutaneous (SC), and visceral (VIS) adipose explants; *b*) examine whether they exert direct effects on isolated mature adipocytes; *c*) determine the effects of an ERα/ERβ antagonist [ICI182,780 (ICI)] on adiponectin release; and *d*) compare the expression of *ER*α, *ER*β, *GPR30*, *ERR*α, *ERR*β, and *ERR*γin breast, SC, and VIS adipose tissue.

## Materials and Methods

### Subjects

The study was approved by the Institutional Review Board of Christ Hospital (Cincinnati, Ohio). Surgical samples were obtained from patients who gave written informed consent. Three types of adipose specimens were obtained: *a*) samples from breast reduction, *b*) abdominal SC samples from abdominoplasty, and *c*) matched VIS (omental) and SC samples from morbidly obese individuals undergoing gastric bypass surgery.

### Explant preparation and incubation

We cut tissue into small (~ 2 × 2 × 2 mm) explants and placed them into 48-well polystyrene plates (70–100 mg/250 μL, four to six wells per treatment) containing glucose- and phenol red-free Dulbecco’s modified Eagle medium supplemented with 10 mM HEPES, 2 mM glutamine, 2 mM pyruvate, and 1% charcoal-stripped fetal bovine serum (Hyclone, Logan, UT). We prepared stock solutions of E_2_ and BPA (Sigma, St. Louis, MO; purity > 99%) and ICI (Tocris, Ellisville, MO) in ethanol at 50–100 mM. Solvent controls (≤ 0.001% ethanol) were included in all experiments. At the end of a 6-hr incubation, explant weights were determined and conditioned media (CM) were collected.

### Cell harvesting and incubation

We used SC adipose tissue from abdominoplasty to prepare mature adipocytes as described by McFarland-Mancini et al. (2006). Briefly, we placed tissue fragments into Hank’s balanced salt solution containing 2% fatty-acid–free bovine serum albumin (BSA) and 200 nM adenosine (to prevent cell rupture). After adding 200 units/g of type IV collagenase (Worthington, Lakewood, MO), we carried out digestion at 37°C. The digest was filtered through a 150-μm mesh and the floating mature adipocytes were separated from the stromal vascular fraction by centrifugation. Adipocytes (100 μL of packed cells) were placed in wide-mouth polypropylene tubes and incubated for 6 hr in the above media containing the various treatments.

### Adiponectin enzyme-linked immunosorbant assay (ELISA)

Adiponectin in CM was quantified by a fluorescent-sandwich ELISA, optimized in our laboratory using a matched monoclonal antibody pair against human adiponectin (MAB10651 capture and BAM1065 biotinylated detection; R&D, Minneapolis, MN). These antibodies recognize epitopes in the globular head of adiponectin and detect all isoforms. Black 96-well plates (Maxisorp; Nunc, Rochester, NY) were coated with the capture antibody and blocked with 0.5% BSA. Plates were then coincubated with the detection antibody and recombinant human adiponectin (R&D) or CM aliquots. After 2 hr, we added streptavidin-conjugated horseradish peroxidase and a fluorimetric substrate (Quantablue; Pierce, Rockford, IL). We read fluorescence at 325 nm excitation and 420 nm emission, using a Gemini XPS fluorescent microplate reader (Molecular Devices, Sunnyvale, CA). The lowest detectable level was 100 pg/mL. We validated assay parameters against commercial plates from the same vendor.

### Real-time polymerase chain reaction (PCR)

We isolated total RNA from breast, VIS, and SC adipose tissue, each pooled from four or five women, followed by synthesis of oligo-dT–primed polyA cDNA as previously described (Hugo et al. 2006). We performed quantitative real-time PCR on 200 ng of cDNA using intron-spanning primers for the various genes listed in Table 1, using Immolase heat-activated Taq DNA polymerase (Bioline, Tauton, MA), and SYBR Green I (Invitrogen, Carlsbad, CA) on a SmartCycler I (Cepheid, Sunnyvale, CA). Cycle parameters were 96°C for 6 min followed by 40 cycles of 95°C for 15 sec, 57°C for 15 sec, and 72°C for 25 sec. We confirmed product purity by melting curve analysis. Each sample was run three times. Changes in gene expression were calculated from the cycle threshold, after correcting for cDNA amounts using β2 microglubulin (*B2M*) expression (Pfaffl et al. 2002). Data are expressed as fold changes over control, which was arbitrarily defined as gene expression in VIS tissue.

### Data analysis

When appropriate, values are expressed as the mean ± SE. We performed statistical analysis using either Student’s *t*-test or one-way analysis of variance followed by Fisher least significant difference post hoc analysis. *p*-Values < 0.05 are considered significant.

## Results

### Suppression of adiponectin release from breast adipose explants by BPA and E_2_

Both adiponectin (Martin et al. 2006) and BPA (Kuruto-Niwa et al. 2007) are detectable in human breast milk. Therefore, we first examined whether BPA alters adiponectin release from breast adipose explants obtained from eight women undergoing breast reduction. As detailed in Table 2, the average age was 43.6 years, and the average body mass index (BMI) was 27, with one woman in the obese category (BMI > 30), four in the overweight category (BMI = 25–30), and three in the normal weight range (BMI ≤ 25). Table 2 also demonstrates the high variability of basal adiponectin release *in vitro*, which showed no apparent relationship to either age or BMI.

**Figure 1:**
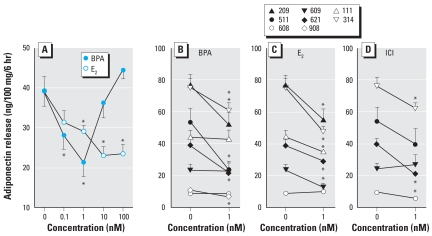
Suppression of adiponectin release from breast adipose explants by BPA, E_2_, and ICI. (*A*) Typical dose response by explants from one patient; each value is the mean ± SE of six determinations. (*B*–*D*) Responses of explants from eight women to 1 nM BPA (*B*), E_2_ (*C*), or ICI (*D*), illustrating variation among patients in both basal adiponectin secretion (see also Table 2) and responsiveness to the test compounds. ^*^p < 0.05 compared with control.

Figure 1A depicts the suppressive effects of both BPA and E_2_ on adiponectin release from breast explants from one patient, selected as a representative. E_2_ showed dose-dependent inhibition of adiponectin release, which was significant (*p* < 0.05) at all doses except 0.1 nM. On the other hand, BPA generated a clear U-shaped response, being significantly suppressive at both the 0.1 and 1 nM doses but not at higher doses. Figure 1B–D shows adiponectin release in response to 1 nM BPA, E_2_, or ICI in explants from individual patients. Suppression of adiponectin by BPA and E_2_ was significant in five of eight and five of six samples tested, respectively. We also examined several samples for the effects of 1 nM ICI. In this case, three of five samples showed significant inhibition.

### BPA at low doses suppresses adiponectin release from abdominal SC explants

We next explored the effects of BPA and E_2_ on adipose tissue other than the breast. For that, we obtained SC abdominal adipose samples from nine women undergoing abdominoplasty. Table 3 shows that the average age was 40.3 years (range, 29–45 years). Five patients had BMI at the normal range, whereas four were in the overweight category. Similar to what we observed in breast explants (Table 2), basal adiponectin release *in vitro* was highly variable, ranging from 7.1 ng/100 mg/6 hr in one patient to 155.2 ng/100 mg/6 hr in another.

**Figure 2:**
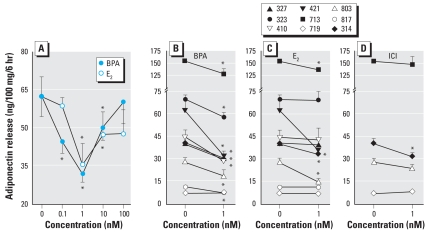
Suppression of adiponectin release from abdominal SC adipose explants by BPA, E_2_, and ICI. (*A*) Typical dose response by explants from one patient; each value is the mean ± SE of six determinations. (*B*–*D*) Responses of explants from nine women to 1 nM BPA (*B*), E_2_ (*C*), or ICI (*D*), illustrating variation among patients in both adiponectin secretion and responsiveness to the test compounds. ^*^p < 0.05 compared with control.

Figure 2A shows the effects of increasing doses of BPA and E_2_ on adiponectin release in an SC abdominal sample from one patient, selected as a representative. Both compounds generated U-shaped curves, with BPA significantly inhibiting adiponectin at the 0.1, 1, and 10 nM doses, whereas E_2_ was effective at the 1 and 10 nM doses. Figure 2B–D shows data from individual patients. BPA at the 1 nM dose significantly inhibited adiponectin in eight of nine samples, whereas E_2_ was effective only in four of nine samples. We examined the effect of 1 nM ICI in four samples, only one of which showed significant inhibition.

### BPA and E_2_ exert direct inhibitory effects on mature adipocytes

**Figure 3:**
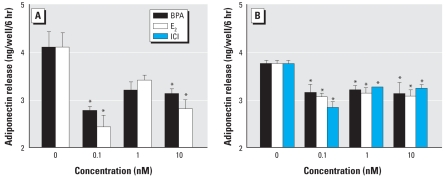
BPA and E_2_ suppress adiponectin release from mature abdominal SC adipocytes from a non-obese woman (*A*) and an obese woman (*B*). (*A*) Effect of treatment with increasing doses of BPA or E_2_. (*B*) Effect of treatment with increasing doses of BPA, E_2_, and ICI. Each value is the mean ± SE of four determinations. ^*^p < 0.05 compared with control.

In addition to mature adipocytes, adipose tissue contains pre-adipocytes, fibroblasts, endothelial cells, and macrophages, many of which affect the secretory activity of the adipocytes (Fain et al. 2004). Thus, we opted to examine if the above compounds have a direct or an indirect effect on adiponectin release. We isolated mature SC adipocytes from several additional women undergoing abdominoplasty. Figure 3 illustrates the secretory profile of adiponectin from a nonobese patient (Figure 3A; 57 years of age, BMI = 28.8) and an obese patient (Figure 3B; 54 years of age, BMI = 45.2). BPA and E_2_ significantly inhibited adiponectin release from mature adipocytes at most doses examined, albeit without exhibiting dose-dependent effects. ICI at all doses examined significantly inhibited adiponectin release (Figure 3B).

### BPA and E_2_ inhibit adiponectin release by SC and VIS explants from morbidly obese patients

**Figure 4:**
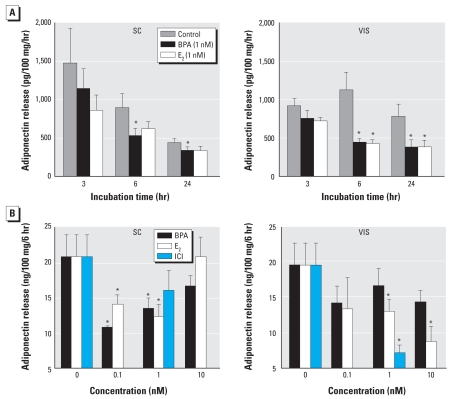
Effects of BPA, E_2_, or ICI on adiponectin release. (*A*) Time-dependent effect of 1 nM BPA or E_2_ on adiponectin release from SC and VIS (omental) adipose tissue explants from a morbidly obese woman. (*B*) Effect of treatment with increasing doses of BPA and E_2_, as well as 1 nM ICI, on adiponectin release from matched abdominal SC and VIS (omental) adipose tissue explants from a morbidly obese man. Each value is the mean ± SE of six determinations. ^*^p < 0.05 compared with control.

To examine whether adiponectin responsiveness to BPA or E_2_ is influenced by obesity, we obtained matched VIS (omental) and SC adipose samples from several morbidly obese patients undergoing gastric bypass surgery. Figure 4A shows results with tissue explants from an extremely obese woman (29 years of age, BMI = 84.5). To compare the rate of adiponectin release over time, in this case we present the data as picograms adiponectin/100 mg/hr. Basal adiponectin release from SC explants showed a time-dependent decline, which was not observed in VIS explants. The time-dependent decline in adiponectin was not due to loss of tissue viability, as determined by the use of a fluorescent Resazurin reduction assay (data not shown). BPA at 1 nM significantly inhibited adiponectin release from SC explants by 50% at 6 hr and 23% at 24 hr, whereas inhibition by E_2_ did not reach statistical significance. We saw a more profound inhibition of 65% and 50% by both BPA and E_2_ in VIS explants at 6 and 24 hr, respectively.

Matched VIS (omental) and SC explants, obtained from a morbidly obese man (54 years of age, BMI = 45.2), were incubated for 6 hr with different doses of BPA, E_2_, and ICI. Figure 4B shows that both BPA and E_2_ were effective in suppressing adiponectin release from SC explants at 0.1 and 1 nM. E_2_ at 1 and 10 nM significantly suppressed adiponectin release from VIS explants, whereas BPA had no effect at all doses examined. Surprisingly, 1 nM ICI suppressed adiponectin release from VIS explants by as much as 70% but had no effect on SC explants.

### Comparison of receptor expression in breast, VIS (omental), and SC adipose tissue

**Figure 5:**
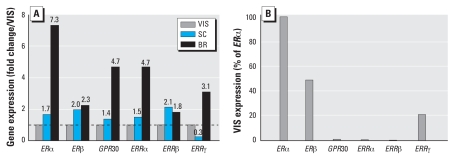
Depot-specific differences in the expression of putative receptors that may mediate the action of BPA or E_2_, as determined by real-time reverse transcriptase PCR. (*A*) Differences in expression of *ER*α, *ER*β, *GPR30*, *ERR*α, *ERR*β, and *ERR*γ in SC and breast (BR) adipose tissue calculated as fold change (shown above bars) relative to VIS adipose tissue. (*B*) Relative abundance of the above receptors in VIS adipose tissue compared with *ER*αexpression.

We next examined breast, VIS, and SC adipose tissue, each pooled from four or five women, for expression of putative receptors that may mediate the actions of BPA and/or E_2_. Figure 5A shows relative mRNA expression of *ER*α, *ER*β, *GPR30*, *ERR*α, *ERR*β, and *ERR*γ in breast and SC adipose tissue, compared with VIS adipose tissue, which was used as a reference. All six receptors were more highly expressed in breast adipose tissue (from 1.8- to 7.3-fold) than VIS adipose tissue. The expression of *GPR30* and *ERR*α was approximately the same in VIS and SC adipose tissue (1.4- to 1.5-fold), whereas *ER*α, *ER*β, and *ERR*β were moderately higher (from 1.7- to 2.1-fold) in SC tissue. Notably, expression of *ERR*γ was much lower (0.3-fold) in SC than in VIS adipose tissue.

Figure 5B shows the relative abundance of mRNA levels of the above receptors in VIS adipose tissue, with expression of the most abundant receptor (*ER*α) presented as 100%. Expression levels of *ER*β and *ERR*γ were 50% and 20%, respectively, relative to *ER*α. On the other hand, expression of *ERR*α, *ERR*β, and *GPR30* was < 1% of *ER*α, indicating a significantly lower abundance.

## Discussion

This study provides the first evidence that BPA at environmentally relevant doses inhibits a key adipokine that protects humans from the sequelae of the metabolic syndrome. BPA at low nanomolar concentrations suppressed adiponectin release from human adipose tissue explants as well as from isolated mature adipocytes. Despite a substantial variability among patients, BPA was as effective, and often more effective, than equimolar concentrations of E_2_. The suppressive effects of BPA were not confined to one adipose tissue type but were present in all depots examined: breast, SC, and VIS. We also report for the first time similar mRNA expression levels of *ER*α, *ER*β, and *ERR*γ in VIS adipose tissue. The expression of *ER*α, *GPR30*, *ERR*α, and *ERR*γ was higher in breast than in either VIS or SC fat. The relative expression of these receptors in VIS adipose tissue was *ER*α > *ER*β > *ERR*γ >>> *GPR30* = *ERR*α = *ERR*β. The role of any of these receptors in mediating the suppressive actions of BPA or E_2_ on adiponectin release remains to be determined.

Previous studies on direct actions of BPA on rodent adipocytes have used very high doses. Sakurai et al. (2004) reported that BPA stimulated insulin-dependent glucose uptake and increased expression of the glucose transporter (Glut4) in 3T3-F442A murine adipocytes, whereas E_2_ was ineffective and ICI did not antagonize BPA. However, only the highest BPA dose (100 μM) was effective. Masuno et al. (2002, 2005) reported that BPA accelerated adipogenesis in 3T3-L1 adipocytes and increased the activity of lipoprotein lipase. Again, BPA was active only at doses of > 80 μM. These data should be interpreted with caution, given the nonlinear dose response of BPA and the potential toxic, or near toxic, levels of BPA. A U-shaped dose–response curve is well recognized for many hormones and toxic compounds, but there is no ready explanation for this phenomenon (Calabrese and Baldwin 2001).

To support the premise that BPA has adverse metabolic effects in humans, it is essential to study its actions on human tissues. Whereas the value of live rodents and murine adipocyte cell lines as experimental models is undisputed, adipocyte biology is sufficiently different between rodents and humans to warrant prudence (Ben Jonathan et al. 2008). For example, the regional distribution of fat depots, their cellular composition (e.g., brown vs. white fat, infiltration by macrophages), and the regulation of resistin, agouti protein, adipsin, and adrenergic receptors are dissimilar in rodents and humans. Intrinsic differences between the species are also exemplified by the suppression of adiponectin expression in 3T3-L1 cells by insulin but its increase in response to insulin in isolated human adipose tissue (Whitehead et al. 2006).

Basal adiponectin release *in vitro* and its responsiveness to BPA or E_2_ were highly variable among patients. This variability results from the combined effects of genetic, nutritional, and hormonal factors, as well as the state of obesity, clinical conditions, and history of drug use. Because all but one of the patients were women, we did not determine the effect of sex. Serum adiponectin levels are moderately higher in women than in men, but hormone replacement therapy does not alter adiponectin release in either pre- or postmenopausal women (Sieminska et al. 2005). The difference in circulating adiponectin between sexes is believed to be due to its suppression by androgens, as supported by an inverse relationship between serum testosterone and adiponectin levels during puberty in men (Andersen et al. 2007). An inadvertent exposure of men to exogenous estrogen-like compounds such as BPA may cause additional suppression of adiponectin, leading to potential harmful consequences. The same concern is extended to prepubertal girls and postmenopausal women with low serum estrogen levels.

Given the relatively small sample size in each category and the observed variability, our data do not lend themselves to definitive conclusions with regard to the relative effectiveness of BPA versus E_2_, which adipose depot is more responsive, whether obesity alters tissue responsiveness, or the potential effects of age. Therefore, we highlight only the general trends observed in this study. For example, BPA, E_2_, and ICI appear to display similar efficacy in suppressing adiponectin release from breast explants, whereas BPA was more effective than E_2_ or ICI in SC adipose explants. In one obese woman, BPA was more effective in suppressing adiponectin from VIS than from SC explants, whereas the reverse was true in an obese man (Figure 4). Recruitment of a larger number of patients will be most helpful in sorting out the effects of age, sex, obesity, or clinical conditions on adipose tissue responsiveness to BPA and/or E_2_.

Most research to date on the biological actions of estrogens has focused on *ER*α. Studies with knockout mice revealed that deletion of *ER*α causes a more severe phenotype than deletion of *ER*β (Couse and Korach 1999). With the exception of few tissues such as the ovary, prostate, and certain brain areas, *ER*α is more highly expressed than *ER*β. Therefore, it was unexpected that human VIS fat expressed similar mRNA levels of both receptors. Using real-time PCR, others reported predominance of *ER*α over *ER*β in isolated mature adipocytes, although *ER*β expression was higher in adipocytes from women than from men (Dieudonne et al. 2004). Given adipose tissue heterogeneity, it is difficult to compare receptor expression in whole adipose tissue, as we used in our studies, with that in isolated adipocytes. In addition, at least four different *ER*β subtypes are expressed in human adipose tissue (Pedersen et al. 2001), with our primers detecting only the common isoform.

The finding that both BPA and E_2_ suppress adiponectin release does not constitute a proof that they act by the same mechanism. In fact, their equipotency strongly suggests involvement of receptors other than classical ERs. The effects of ICI further confound the issue. In these studies, ICI at low doses either suppressed or had no effect on adiponectin release. In samples pretreated with ICI before exposure to BPA or E_2_, we observed neither blockade of suppression nor additive effects (data not shown). Thus, in terms of the control of adiponectin release, ICI does not behave as a typical ERα/ERβ antagonist. The suppressive effect of ICI also differentiate the putative receptor in human adipose tissue from the ncmER reported by Alonso-Magdalena et al. (2005) that is activated rapidly and is unresponsive to ICI. Although searching for potential mechanisms for the actions of BPA and E_2_, we examined published values of their binding affinity to several putative receptors. Although BPA has a lower median effective concentration (EC_50_) for ERβ than for ERα (Kuiper et al. 1998), it is still in the micromolar range, compared with a low nanomolar range for E_2_. On the other hand, the EC_50_ for BPA for GPR30 is 630 nM (Thomas and Dong 2006) and is as low as 8.9 nM for ERRγ(Okada et al. 2008).

GPR30 is a seven-transmembrane receptor that increases the activity of second messengers such as adenylate cyclase and mitogen-activated protein kinase in response to E_2_ in ER-negative breast cancer cell lines (Filardo and Thomas 2005). Notably, the ER antagonist ICI functions as a GPR30 agonist. Our data are the first to show expression of *GPR30* in human adipose tissue, albeit at very low abundance compared with either *ER*α or *ER*β (Figure 5). Another potential candidate is *ERR*γ, whose expression level in VIS adipose tissue was 4- to 5-fold lower than that of *ER*α and *ER*β. The ERRs are orphan nuclear receptors that are constitutively active and do not bind estrogens (Ariazi and Jordan 2006). *ERR*γ is expressed in a tissue-specific manner (Heard et al. 2000), but little is known about its biological functions. Future studies should confirm expression of these receptors at the protein level and then use small interfering RNA to determine the consequences of receptor knockdown on the suppressive effects of E_2_ or BPA on adiponectin release. It would also be of interest to examine whether BPA at low doses affects adipogenesis, lipogenesis/lipolysis, or the release of other adipokines.

## Conclusion

The growing interest by scientists and the public alike in BPA has placed this compound at the center of the debate over potential adverse effects of man-made chemicals found in the environment on fetal/neonatal development, reproductive fecundity, metabolic homeostasis, and carcinogenesis. Yet, attribution of such actions to BPA has been controversial. Differences of opinion and disagreements over data interpretation underlie the inability of several expert panels, convened periodically since 1999, to convince regulatory agencies that BPA poses hazards to human health. There is a growing recognition that the roles of genetic predisposition and environmental factors in the epidemic of obesity and related diseases should not be ignored. Given the endurance of BPA in the environment, its presence in serum from humans worldwide, and the suppression of adiponectin release at nanomolar concentrations, BPA may indeed be the bona fide endocrine disruptor that adversely affects metabolic homeostasis and its manifestations.

## Figures and Tables

**Table 1:** Human gene-specific primers for quantitative real-time reverse transcriptase PCR.

Gene	Accession no.^a^	Forward primer (5′→3′)	Reverse primer (5′→3′)	Product size (bp)
*ESR1*	NM_000125	CAGGCACATGAGTAACAAAGG	CAAGGAATGCGATGAAGTAGAG	195
*ESR2*	NM_001437	CAGTTATCACATCTGTATGCGG	ACTCCATAGTGATATCCCGA	208
*ESRRA*	NM_004451	ACTGCAGGATGAGCTGG	TGCACAGAGTCTGAATTGG	185
*ESRRB*	NM_004452	CTGGTGTACGCTGAGGA	TACATGGAATCGGAGTTGG	172
*ESRRG*	NM_001438	CATATTCCAGGCTTCTCCA	GACAAGTTCATCCTCAAACGA	122
*GPR30*	NM_001039966	ACGAGACTGTGAAATCCGCAACCA	ATCAGGCTGGAGGTGCACTTGGAA	153
*B2M*	NM_004048	GGCATTCCTGAAGCTGAC	GAATCTTTGGAGTACGCTGG	114

Primer pairs are all intron-spanning pairs. Abbreviations: *ESR1*, ERα; *ESR2*, ERβ; *ESRRA*, ERRα; *ESRRB*, ERRβ; *ESSRG*, ERRγ(all three transcripts); *B2M*, β2-microglobulin.

a GenBank accession numbers (National Center for Biotechnology Information 2008).

**Table 2:** Breast explants by identification number (ID), patient’s age, BMI (kg/m^2^), and basal *in vitro* adiponectin release (Adipo).

ID	Age (years)	BMI	Adipo^a^
209	51	28.2	77.3
511	30	36.6	53.5
608	57	25.2	8.6
609	40	28.1	23.6
621	23	21.5	39.1
908	57	26.9	10.4
111	58	22.5	44.4
314	33	27.3	75.5
Mean ± SE	43.6 ± 4.9	27.0 ± 1.6	41.6 ± 9.4

a ng/100 mg/6 hr.

**Table 3:** Abdominal SC explants by identification number (ID), patient’s age, BMI (kg/m^2^), and basal *in vitro* adiponectin release (Adipo).

ID	Age (years)	BMI	Adipo^a^
327	37	24.8	40.7
323	42	24.8	70.0
410	44	24.4	44.7
421	45	20.9	62.5
713	45	21.4	155.2
719	44	28.3	7.1
803	44	26.1	28.2
817	29	26.3	11.6
314	33	27.3	40.4
Mean ± SE	40.3 ± 2.0	24.9 ± 0.8	51.2 ± 14.7

a ng/100 mg/6 hr.
